# Regulation of the NRF2 transcription factor by andrographolide and organic extracts from plant endophytes

**DOI:** 10.1371/journal.pone.0204853

**Published:** 2018-10-01

**Authors:** Daphne Pei Wen Wong, Mei Ying Ng, Jia Yu Leung, Boon Kim Boh, Ee Chien Lim, Shi Hua Tan, Shuying Lim, Wen Hui Seah, Christine Zhiwen Hu, Boon Chuan Ho, Daphne Hui Ping Ng, Thilo Hagen

**Affiliations:** 1 Department of Biochemistry, Yong Loo Lin School of Medicine, National University of Singapore, Singapore, Singapore; 2 National Heart Centre Singapore, Singapore, Singapore; 3 The Herbarium, Singapore Botanic Gardens, National Parks Board, Singapore, Singapore; 4 School of Civil and Environmental Engineering, Nanyang Technological University, Singapore, Singapore; George Washington University, UNITED STATES

## Abstract

The transcription factor NF-E2 Related Factor-2 (NRF2) is an important drug target. Activation of NRF2 has chemopreventive effects in cancer and exerts beneficial effects in a number of diseases, including neurodegenerative diseases, inflammatory diseases, hepatosteatosis, obesity and insulin resistance. Hence, there have been great efforts to discover and characterize novel NRF2 activators. One reported NRF2 activator is the labdane diterpenoid andrographolide. In this study, we identified the mechanism through which andrographolide activates NRF2. We showed that andrographolide inhibits the function of KEAP1, a protein that together with CUL3 and RBX1 forms an E3 ubiquitin ligase that polyubiquitinates NRF2. Andrographolide partially inhibits the interaction of KEAP1 with CUL3 in a manner dependent on Cys151 in KEAP1. This suggests that andrographolide forms Michael acceptor dependent adducts with Cys151 in KEAP1 *in vivo*, leading to inhibition of NRF2 ubiquitination and consequently accumulation of the transcription factor. Interestingly, we also showed that at higher concentrations andrographolide increases NRF2 protein expression in a Cys151 independent, but likely KEAP1 dependent manner, possibly through modification of other Cys residues in KEAP1. In this study we also screened secondary metabolites produced by endophytes isolated from non-flowering plants for NRF2-inducing properties. One of the extracts, ORX 41, increased both NRF2 protein expression and transcriptional activity markedly. These results suggest that endophytes isolated from non-flowering or other plants may be a good source of novel NRF2 inducing compounds.

## Introduction

Plant extracts are known to have anti-inflammatory properties and are widely used in traditional medicine. Andrographolide, a labdane diterpenoid, was isolated from the popular medicinal plant *Andrographis paniculata*. This herb has long been used as traditional medicine in Asian countries for the treatment of the common cold, sore throat, upper respiratory tract infections, fever, diarrhea and inflammation [1]. Andrographolide has been reported to exhibit anti-cancer and anti-inflammatory properties both *in vitro* and *in vivo* [2] and may have neuroprotective potential in the treatment of stroke [3].

**Fig 1:**
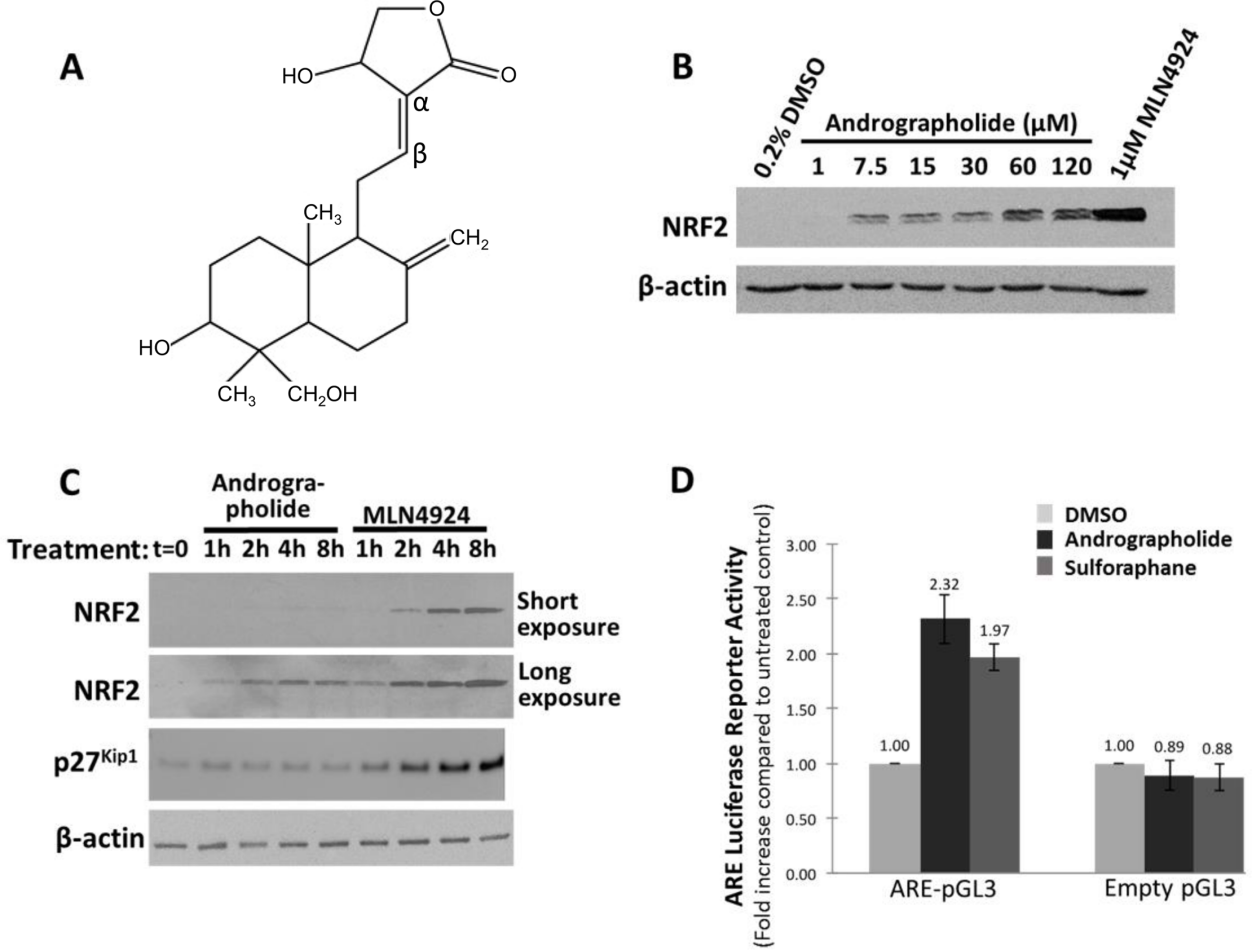
Andrographolide increases NRF2 protein expression and transcriptional activity. (A) Structure of andrographolide. The α and β carbons of the Michael acceptor group are indicated. (B) HEK293T cells were treated with increasing andrographolide concentrations, as indicated, for 4 hours. DMSO was added as a negative control and the Cullin E3 RING ligase (CRL) inhibitor MLN4924 (1 μM) served as positive control. Cells were lysed and cell lysates analyzed by Western blotting using NRF2 and actin antibodies. (C) To measure the time dependence of andrographolide dependent NRF2 activation, HEK293T cells were treated with 7.5 μM andrographolide or 1 μM MLN4924 for the indicated times. Subsequently, cell lysates were analyzed by Western blotting with the indicated antibodies. The CRL substrate p27^Kip1^ served as a positive control for the CRL inhibitor MLN4924. (D) To measure Nrf2 transcriptional activity, the ARE luciferase reporter assay was performed as described under Materials and Methods. The cells were transfected for 24 hours before treatment with andrographolide (7.5 μM) or sulforaphane (10 μM) for 6 hours. The results shown are representative of two independent experiments.

Mechanistically, andrographolide has been shown to decrease inflammation through the inhibition of NF-κB activation [4]. Andrographolide contains a Michael acceptor group. The carbon in the β-position from the carbonyl moiety of the Michael acceptor group can form a covalent bond with the thiolate group of reactive cysteine residues (Fig 1A). Xia et al., 2004 have shown that andrographolide covalently reacts with cysteine 62 of the p50 subunit of NF-κB, accounting for the inhibitory effect on NF-κB transcriptional activity.

Apart from the regulation of NF-κB signaling, it has been reported in a number of studies that andrographolide also functions as an activator of the NF-E2 Related Factor-2 (NRF2) [5]. NRF2 is a basic leucine zipper (bZIP) transcription factor that regulates the expression of phase I drug metabolizing enzymes and antioxidant proteins. Drugs that activate NRF2 are studied for the treatment of diseases that are associated with oxidative stress, such as neurodegenerative diseases. NRF2 activators are also considered as chemopreventive agents in cancer. Thus, by activating NRF2, andrographolide exerts cytoprotective effects.

It was recently reported that andrographolide upregulates NRF2 in a manner dependent on p38 MAPK and ERK activation [6, 7]. Andrographolide has also been reported to from adducts with Cys77, Cys151, Cys273 and Cys368 in KEAP1 [8]. The KEAP1 protein is the major negative regulator of NRF2. KEAP1 is part of an E3 ubiquitin ligase complex that also contains the scaffold protein Cullin 3 (CUL3) and the RING domain containing protein RBX1 [9]. In this complex, KEAP1 functions as a substrate recognition subunit. KEAP1 binds via its double-glycine repeat (DGR) domain to the NRF2 substrate and via its ‘‘Bric a brac, Tramtrack and Broad Complex/Pox virus and Zinc finger” (BTB/POZ) domain to the CUL3 scaffold. The RBX1 subunit recruits ubiquitin charged E2 ubiquitin conjugating enzymes to facilitate the transfer of ubiquitin onto lysine residues in NRF2. Various NRF2 activating drugs function by forming adducts with cysteine residues in KEAP1. One major cysteine that is modified by a number of NRF2 activating compounds, such as sulforaphane and the tripertinoid CDDO-Me, is Cys151. It has been reported that modification of Cys151 by these compounds leads to the dissociation of KEAP1 from CUL3, thus preventing NRF2 ubiquitination [10–14].

Here we hypothesized that andrographolide could induce NRF2 by forming a covalent adduct with critical cysteine residues in KEAP1 [8], resulting in the inhibition of KEAP1-dependent NRF2 degradation. We found that the induction of NRF2 by andrographolide is dependent on the presence of Cysteine 151 in KEAP1, strongly implicating this residue as the major molecular target of andrographolide. Andrographolide induced KEAP1 modification at Cys151 leads to partial dissociation of KEAP1 from CUL3, thus inhibiting NRF2 ubiquitination and degradation, and ultimately leading to the induction of the phase 2 response. Of note, at higher concentrations, andrographolide also induces NRF2 protein expression in a Cys151 independent manner.

NRF2 can also be induced by various other plant derived compounds [15]. Such bioactive metabolites may either be produced by the plants themselves or by endophytes living within the plant tissues [16]. In this study, secondary metabolites produced by endophytes isolated from non-flowering plants were screened for Nrf2-inducing properties. We focussed specifically on non-flowering medicinal plants, particularly tropical ferns and mosses that are local to Singapore, given that these plants are less studied as endophyte reservoirs. Among the plants chosen were the tropical ferns *Phlegmariurus phlegmaria*, *Taenitis blechnoides*, *Pyrrosia piloselloides*, *Psilotum nudum*, *Ophioglossum reticulatum* and *Dicranopteris linearis* as well as the mosses *Calymperes erosum*, *Campylopus serratus*, *Philonotis thwaitesii* and *Vesicularia dubyana*. Given that a number of these plants have been implicated to have anti-inflammatory and antioxidant properties, we hypothesized that the fungal and bacterial endophytes living within these plants may also produce secondary bioactive metabolites with NRF2 inducing properties. Notably, the extract ORX 41 showed a marked increase in NRF2 protein expression and transcriptional activity compared to the vehicle control. Further studies are needed to identify the exact component within this crude organic extract that accounts for the NRF2 inducing properties. The findings from this study have important implications for the understanding of the anti-inflammatory mechanism of andrographolide as well as the identification of novel plant derived NRF2 inducing compounds.

## Materials and methods

### Collection of tropical ferns and mosses

The tropical ferns (*Dicranopteris linearis*, *Ophioglossum reticulatum*, *Psilotum nudum*, *Pyrrosia piloselloides* and *Taenitis blechnoides*) and mosses (*Calymperes erosum*, *Campylopus serratus*, *Philonotis thwaitesii* and *Vesicularia dubyana*) were collected within the National University of Singapore (NUS) Kent Ridge campus (1° 17' 48.2" N, 103° 46' 34.7" E). The lycophyte *Phlegmariurus phlegmaria* was purchased from a local plant nursery. All species collected from the NUS campus are not listed as threatened. They are also not protected locally or internationally. The specimens were not collected within protected areas or national parks/reserves and are common species that can be found in urban areas throughout Singapore. They were also not deliberately planted. Staff of NUS are granted permission to collect samples within the campus grounds for teaching and research purposes. Hence, no permission was required.

After washing with tap water to remove surface debris, plant parts were cut into approximately 1 cm pieces and surface-sterilized by washing with agitation using 95% ethanol for 10 s, 10% bleach solution for 2 min and 70% ethanol for 2 min. After air-drying, plant segments were transferred onto LB Agar, M2 Agar, 2% Malt Extract Agar, Glycerol-Arginine Agar and Soy Agar as described in [17]. The plated plant cuttings were incubated at room temperature on the solid media and observed over 3 weeks for the emergence of endophytes from the cut ends. Endophytes were isolated and maintained on respective solid media. A total of 49 species of bacterial and fungal endophytes were isolated (data not shown).

### Characterization and identification of endophytes

The isolated endophytes were then characterized. The morphological characterization of the endophytes was first conducted by Gram staining and lactophenol cotton blue staining for bacterial and fungal endophytes, respectively. Bacterial and fungal endophytes were identified by PCR amplification and sequencing of the 16S rDNA and ITS rDNA respectively. The 16S rDNA of bacterial endophytes was PCR amplified using primers 27F (5′-AGAGTTTGATCCTGGCTCAG- 3′) and 1492R (5′-GGTTACCTTGTTACGACTT-3′). PCR amplification of the ITS rDNA of fungal endophytes was performed using forward primer ITS5 (5’-GGAAGTAAAAGTCGTAACAAGG-3’) and reverse primer ITS4 (5’-TCCTCCGCTTATTGATATGC-3’). PCR products were sequenced and the sequences were compared with those available in GenBank via BLASTn searches to identify endophytes.

### Organic extraction of secondary metabolites from endophytes

To extract secondary metabolites from bacterial endophytes, a single colony of the bacterial endophyte was inoculated in 5 ml of culture medium for 16 h in a 37°C shaker. 2 ml of the overnight culture was then sub-cultured in 100 ml of fresh medium for 24 to 72 h for the optimum production of secondary metabolites. An equal volume of dichloromethane was added and the mixture was gently swirled for 1 h at 100 rpm for maximum extraction before it was transferred to a separatory funnel. The mixture was left to separate overnight. The dichloromethane fraction (organic fraction) was collected and dried using a rotary evaporator. 1 ml of methanol was added to each of the dried extracts and stored at -20°C.

To extract secondary metabolites from fungal endophytes, 2 pieces of agar media with 4-week old fungal endophytes were cut into small pieces, immersed in 100 ml dichloromethane and gently swirled for 1 h at 100 rpm. The mixture was left to separate overnight. The dichloromethane fraction was filtered with a cheese-cloth to remove fungal and agar remnants. The dichloromethane fraction (organic fraction) was collected and dried using a rotary evaporator. 1 ml of methanol was added to each of the dried extracts, which were then stored at -20°C.

### Cell culture and transfection

HEK293T cells were cultured in Dulbecco’s Modified Eagle Medium (DMEM) (Invitrogen) supplemented with 10% (vol/vol) heat-inactivated fetal bovine serum (Hyclone), 2 mM L-glutamine (Invitrogen), 100 U/ml penicillin and 100 μg/ml streptomycin (Invitrogen) in a humidified 37°C, 5% CO_2_ tissue culture incubator. Transient transfections were performed using Genejuice transfection reagent (Novagen) in accordance with the manufacturer’s directions for sub-confluent cells. Andrographolide was purchased from Sigma-Aldrich.

### Plasmid constructs

The plasmid constructs, including NRF2-HA-pcDNA3, wild type and mutant FLAG-KEAP1-pcDNA3.1 and V5-KEAP1-pcDNA3.1, CUL3-V5-pcDNA3, CUL3-HA-pcDNA3 [18, 19]. The Antioxidant Response Element (ARE)-luciferase-pGL2 reporter plasmid was a kind gift from Dr. Alan Porter [20]. The reporter plasmid contains one binding site for the NRF2/Maf heterodimeric transcription factor. The plasmid was generated by amplifying the 25bp sequence (GCAGTCACAGTGACTCAGCAGAATC) of the antioxidant response element upstream of the *Nqo1* gene (NCBI# M81596) from SH-Sy5y cells genomic DNA. The amplified product was cloned into the NheI and XhoI sites of pGL2 Promoter (Promega) reporter plasmid. To generate the FLAG-KEAP1 KLHL12 BTB plasmid, a modified pcDNA3.1 backbone with an N-terminal 2xFLAG tag, followed by KpnI and XbaI restriction sites, was used. The KEAP1 KLHL12 BTB insert was cloned into the KpnI and XbaI restriction sites and consisted of the KEAP1 N-terminal (NT) region (KEAP1 amino acids 2–60), followed by the KLHL12 BTB domain (KLHL12 amino acids 33–128) and the KEAP1 intervening region (IVR) and double-glycine repeat domain (DGR) and the C-terminal (CT) region (KEAP1 amino acids 180–624). The plasmids denoted ΔNT lack the KEAP1 N-terminal region (amino acids 1–60).

### Immunoblotting

Cells were washed with ice-cold Phosphate-Buffered Saline (PBS) and then lysed in Triton X-100-containing lysis buffer. The composition of the lysis buffer was as follows: 25 mM Tris-HCl (pH 7.5), 100 mM NaCl, 2.5 mM EDTA, 2.5 mM EGTA, 20 mM NaF, 1 mM Na_3_VO_4_, 20 mM sodium β-glycerophosphate, 10 mM sodium pyrophosphate, 0.5% Triton X-100, Roche protease inhibitor cocktail and 0.1% β-mercaptoethanol. Lysates were precleared by centrifugation before use for Western blotting. Equal amounts of protein were loaded for Western blot analysis. The following antibodies were used: anti-NRF2 (Cell Signaling Technology), anti-p27^Kip1^ (BD Biosciences), anti-α-tubulin (Molecular Probes), anti-β-actin (Sigma), anti-FLAG M2 (Sigma), anti-V5 (Serotec) and anti-HA (Roche).

### Immunoprecipitation

20 μl of anti-FLAG M2 agarose beads (Sigma) or mouse monoclonal V5 antibody (Serotec), coupled to 20 μl of protein G-sepharose beads (Amersham Biosciences), were used for immunoprecipitation. 500 μl of precleared lysate from cells transfected with FLAG- or V5-tagged Keap1 or empty vector in 60-mm tissue culture dishes was added to the beads. The samples were tumbled for 1 h at 4°C, and the beads were then washed four times in ice-cold NP40 lysis buffer (containing 50 mM NaCl, 0.5% NP-40, 5% glycerol, 0.5 mM EDTA, 50 mM Tris, pH 7.5) and once in ice-cold buffer containing 50 mM Tris (pH 7.5). The immunoprecipitated Keap1 proteins were then eluted and denatured in 2X Laemmli sample buffer (Bio-Rad) containing 5% β-mercaptoethanol and subjected to SDS-PAGE and Western blotting.

### Investigation of the effects of organic extracts isolated from the endophytes on Nrf2 transcriptional activity

We screened the 49 organic extracts for NRF2-inducing properties using the ARE dependent luciferase reporter assay. Briefly, cells were cultured in 12-well plates and at approximately 50% confluency, transfected with 0.4 μg ARE-pGL3 plasmid or 0.4 μg empty pGL3 DNA per well for 24 h. Cells were then treated with the compounds or extracts in duplicates. Luciferase activities were assayed using the Steady-Glo Luciferase Assay System (Promega).

## Results

### Andrographolide increases NRF2 transcriptional activity and protein concentration

To confirm the previously reported effect of andrographolide on NRF2 protein concentrations and NRF2 transcriptional activity, we treated HEK293T cells for four hours with increasing concentrations of andrographolide. The Nedd8 E1 inhibitor MLN4924 served as a positive control in this experiment. MLN4924 inhibits the function of all Cullin RING E3 ubiquitin ligases (CRLs), including the CUL3/RBX1/KEAP1 ligase, leading to a marked NRF2 stabilization (Fig 1B). We observed that treatment with andrographolide resulted in a dose-dependent increase in endogenous NRF2 concentrations. The lowest andrographolide concentration that induced a robust response was 7.5 μM, and this concentration was used in further experiments.

**Fig 2:**
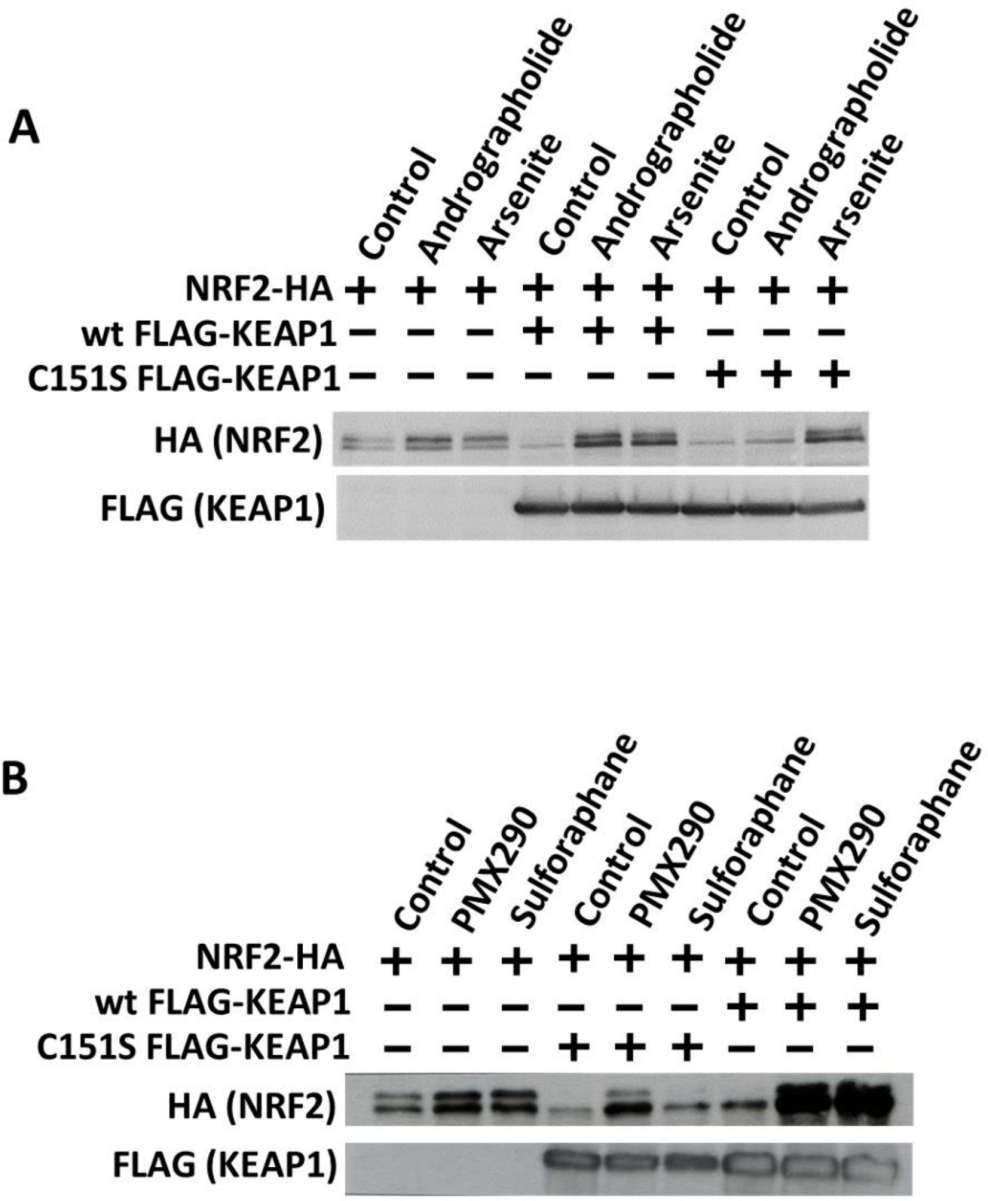
Western blot analysis of NRF2 protein concentrations in HEK293T cells after treatment with andrographolide. (A, B) HEK293T cells were transfected with HA-tagged NRF2 and either wild type FLAG-tagged KEAP1 or C151S KEAP1 mutant for 48 hours before treatment with andrographolide (7.5 μM), arsenite (20 μM), PMX290 (2 μM), or sulforaphane (10 μM) for 6 hours. The cell lysate samples were subjected to SDS-PAGE and analyzed using Western Blotting with HA and FLAG antibodies.

We also found that andrographolide increased NRF2 protein levels in a time dependent manner (Fig 1C, long exposure). Compared to andrographolide, the CRL inhibitor MLN4924 led to a greater induction of NRF2 protein concentrations. However, the time course with which andrographolide and MLN4924 induced NRF2 protein levels was similar (compare the andrographolide dependent NRF2 induction in the long exposure to the MLN4924 dependent induction in the short exposure). This result is consistent with the hypothesis that both andrographolide and MLN4924 function to inhibit NRF2 degradation by inhibiting the CUL3/RBX1/KEAP1 E3 ligase, respectively. In further support, andrographolide also increased the expression of transfected NRF2 (Fig 2A), suggesting that the drug increases NRF2 protein expression via a posttranslational mechanism.

We finally determined the effect of andrographolide on NRF2 transcriptional activity by using an Antioxidant Regulatory Element” (ARE) dependent gene reporter assay. We found that andrographolide induced a similar increase in NRF2 transcriptional activity compared to sulforaphane (Fig 1D). In conclusion, andrographolide increases NRF2 protein expression and transcriptional activity at low micromolar concentrations.

### Andrographolide induces NRF2 in a manner dependent on Cys151 in KEAP1

We then tested the hypothesis that andrographolide induces NRF2 by inhibiting KEAP1 in a Cys151 dependent manner. To this end, we transfected cells with wild type and C151S mutant KEAP1. If andrographolide induces NRF2 through inhibiting KEAP1 via interaction with Cys151, no induction of NRF2 would be expected in cells expressing C151S mutant KEAP1. As shown in Fig 2A, andrographolide induced NRF2 protein expression in cells transfected with empty vector or wild type KEAP1. In contrast, andrographolide was very consistently without effect in cells expressing C151S mutant KEAP1 (Fig 2A, S1 Fig). In contrast to andrographolide, arsenite induced NRF2 protein levels in cells transfected with wild type as well as C151S KEAP1. This indicates that the mechanism of arsenite is Cys151 independent, as previously reported [21]. We also used two other NRF2 inducing compounds, PMX290 [19] and sulforaphane. PMX290 induced NRF2 protein levels in a Cys151 independent manner (Fig 2C), whereas the effect of sulforaphane was Cys151 dependent, consistent with previous reports [12–16, 19, 21, 22]. We conclude that andrographolide induces NRF2 in a manner dependent on Cys151 in KEAP1. Hence, andrographolide appears to have a similar mechanism of action compared to the well characterized NRF2 inducer sulforaphane.

### Effect of andrographolide on the interaction between KEAP1 and CUL3

**Fig 3:**
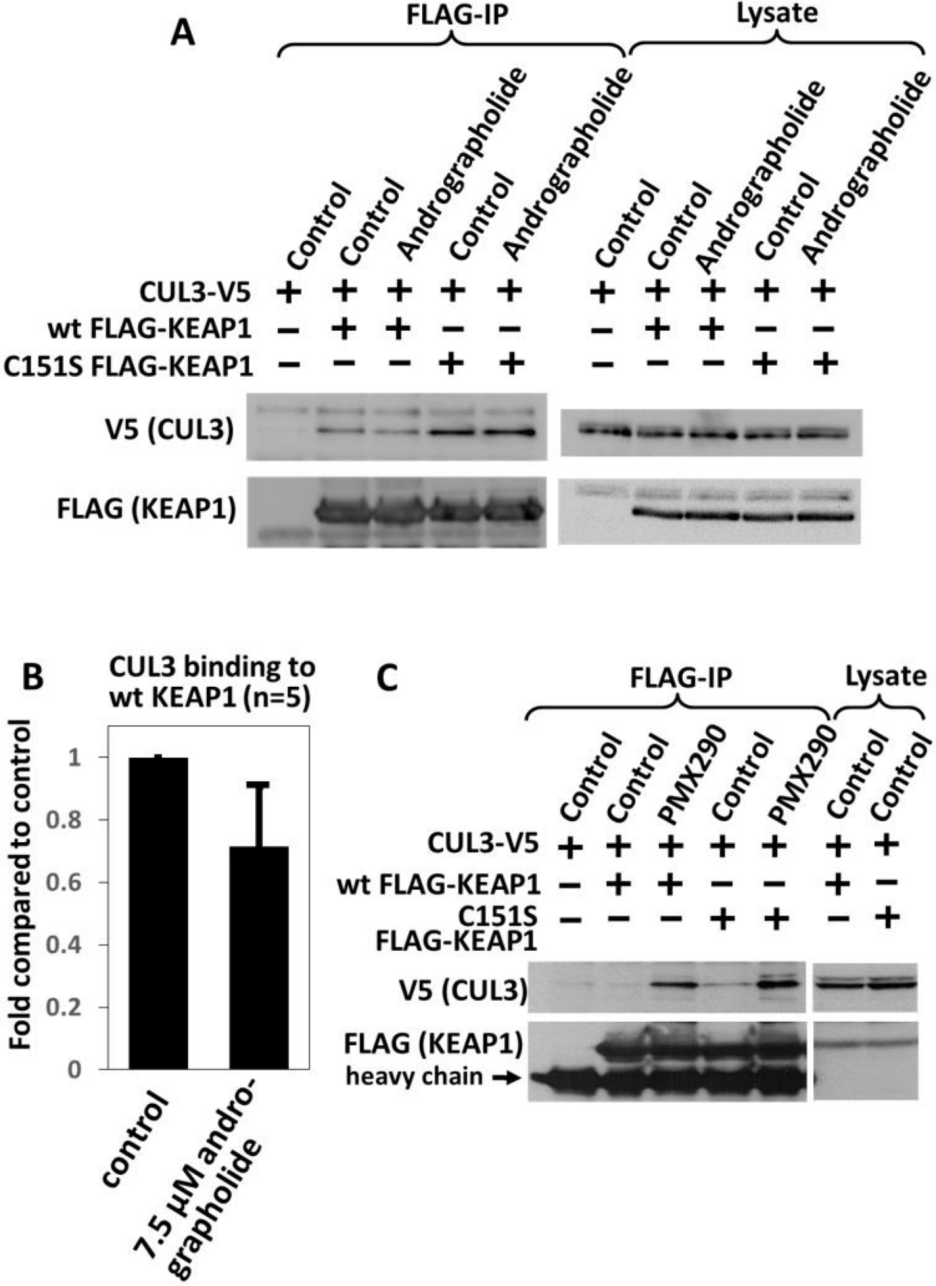
Effect of andrographolide on the interaction between KEAP1 and CUL3. (A, B) The cells were transfected with the indicated expression plasmids for two days, followed by drug treatment with 7.5 μM andrographolide (A) or 2 μM PMX290 (B) for 4 hours or 6 hours, respectively. Cells were then lysed and subjected to FLAG immunoprecipitation, as described under Materials and Methods. The data in (C) represent the mean and S.E.M. of 5 independent repeats of the immunoprecipitation in (A).

Sulforaphane has been shown to disrupt the binding between CUL3 and KEAP1, leading to inhibition of CUL3 dependent ubiquitin transfer onto NRF2 [10]. We hence tested whether andrographolide exerts a similar inhibitory effect on the interaction between CUL3 and KEAP1. As shown in Fig 3A, andrographolide caused indeed a decrease in the binding of CUL3 to KEAP1, although the drug did not abolish the interaction between the two proteins. Densitometry of five independent experiments indicated that andrographolide led to an approximately 30% decrease in the binding between CUL3 and KEAP1 (Fig 3B). Of note, the decrease in the interaction between CUL3 and KEAP1 was dependent on Cys151 in KEAP1 (Fig 3A, S4 Fig).

**Fig 4:**
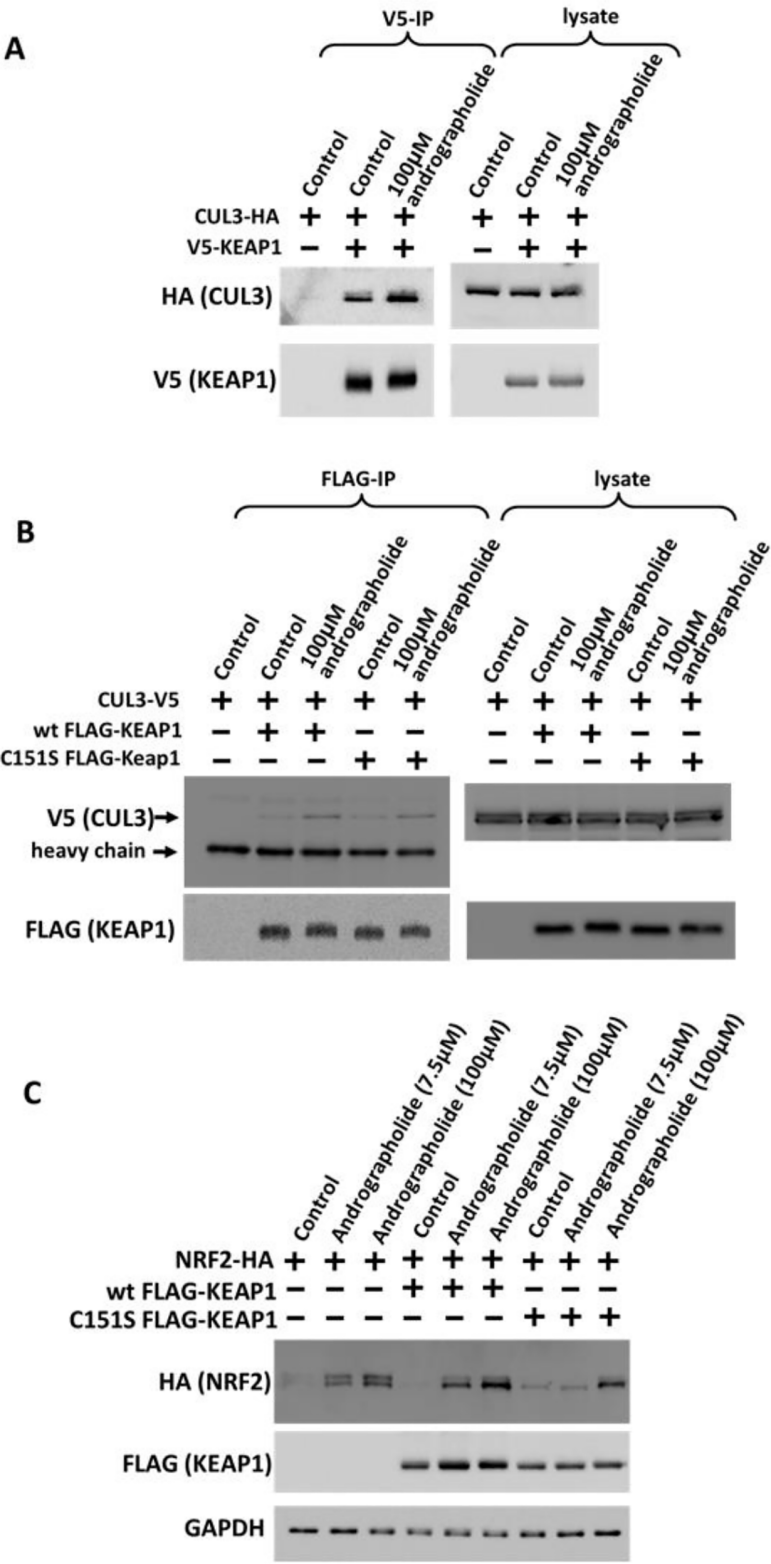
At high concentration, andrographolide functions independently of Cys151 in KEAP1. (A, B) Cells were transfected with the indicated expression plasmids for 2 days, followed by drug treatment with 100 μM andrographolide for 4 hours, as indicated. Cells were then lysed and subjected to immunoprecipitation with V5 antibody, coupled to protein G-sepharose (A) or FLAG M2 agarose (B), as described under Materials and Methods. Cell lysates and immunoprecipitates were analyzed by Western blotting with the indicated antibodies. (C) Cells were transfected with HA-tagged NRF2 and either wild type FLAG-tagged KEAP1 or C151S KEAP1 mutant for two days and then treated with 7.5 μM or 100 μM andrographolide for 4 hours. The cell lysate samples were analyzed using Western Blotting with the indicated antibodies.

The inhibitory effect of andrographolide (used at 7.5 μM) on the interaction between CUL3 and KEAP1 was only moderate. As shown in Fig 1B, an andrographolide concentration of 7.5 μM does not cause maximum NRF2 induction. We hence tested the effect of a higher concentration of andrographolide (100 μM). Surprisingly, at this concentration, andrographolide caused an increase in CUL3-KEAP1 binding (Fig 4A). Similar to the high concentration of andrographolide, PMX290 and arsenite also increased the interaction between CUL3 and KEAP1 (Fig 3C) [19, 21]. PMX290 and arsenite stabilized NRF2 in a manner independent of Cys151 in KEAP1 (Fig 2). We hence tested whether andrographolide also functions in a Cys151 independent manner at higher concentrations. We indeed found that at 100 μM, the andrographolide-induced increase in the CUL3-KEAP1 interaction is independent of Cys151 (Fig 4B). Furthermore, NRF2 induction by andrographolide at a concentration of 100 μM did not require Cys151 in KEAP1 (Fig 4C). We thus conclude that at low concentrations, andrographolide decreases binding between CUL3 and KEAP1 and stabilizes NRF2 in a Cys151-dependent manner. In contrast, at high concentrations, the drug increases CUL3-KEAP1 binding and induces NRF2 in a manner independent of Cys151.

**Fig 5:**
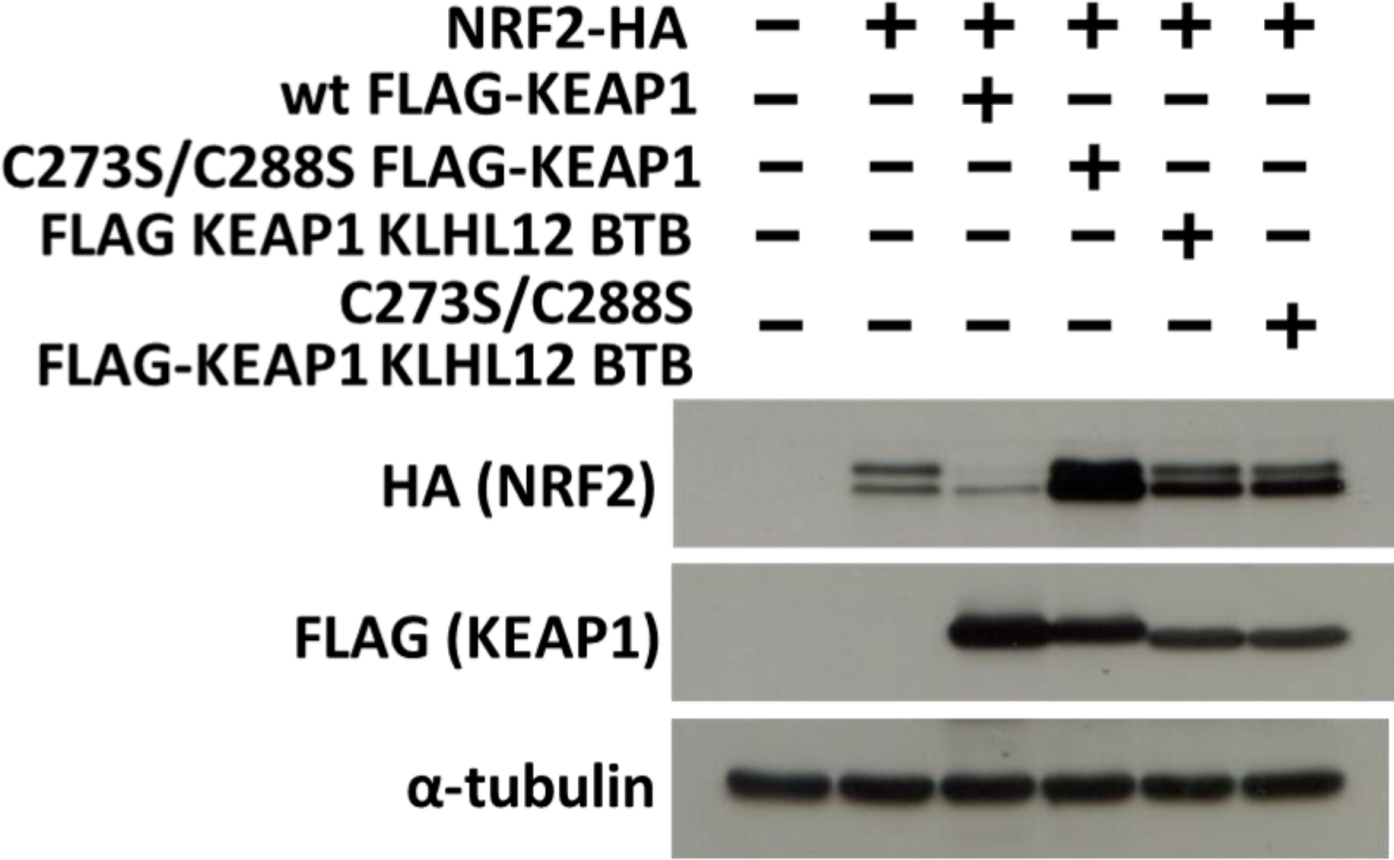
The role of the KEAP1 BTB domain in mediating NRF2 ubiquitination. Cells were transfected with the indicated expression plasmids for 2 days, followed by cell lysis and Western blotting with FLAG, HA and α-tubulin antibodies. Details of the used expression plasmids are included in Materials and Methods.

Cys151 is localized in the BTB domain of KEAP1. Two important functions of BTB domains are to mediate binding to CUL3 as well as to mediate the homodimerization of the CUL3 substrate receptor proteins. As discussed above, modification of Cys151 in the KEAP1 BTB domain inhibits the binding to CUL3. We hence tested whether swapping of the KEAP1 BTB domain with that of another CUL3 substrate receptor (KLHL12) that lacks a homologous cysteine is functional in inducing NRF2 degradation. The BTB domains of KEAP1 and KLHL12 are approximately 50% identical. The KEAP1-KLHL12 BTB chimera showed a similar ability to homodimerize and bind to CUL3 and NRF2 compared to wild type Keap1 (S2 Fig). However, swapping of the BTB domain impaired the function of KEAP1 to induce NRF2 degradation (Fig 5). This suggests that the KEAP1 BTB domain exerts additional conformational control necessary for NRF2 ubiquitination.

On the other hand, both quinols and arsenite induce NRF2 accumulation in a manner independent of Cys151 and increase the binding between CUL3 and KEAP1. One possibility is that quinols and arsenite modify Cys273 and Cys288, two residues that are required for KEAP1 activity. We hence tested the effect of Cys273 and Cys288 mutation on binding of KEAP1 to CUL3. As previously reported, the C273S/C288S mutant was inactive in terms of its ability to induce NRF2 degradation (Fig 5), despite a similar subcellular expression level and intracellular localization compared to wild type KEAP1 (S3 Fig). However, we found that the mutation of Cys273 and Cys288 had no effect on the binding of KEAP1 to CUL3 (or on binding to NRF2 and KEAP1 homodimerization) (S2 Fig). This suggests that quinols and arsenite function by modifying other cysteine residues in Keap1 or that the observed increase in binding between CUL3 and KEAP1 is not involved in the mechanism of action of these drugs.

In conclusion, low concentrations of andrographolide function to stabilize NRF2 in a manner dependent on Cys151 in KEAP1, and this effect may be due to dissociation of CUL3 from KEAP1. At high concentrations, andrographolide stabilizes NRF2 in a Cys151 independent manner and increases the binding between CUL3 and KEAP1. However, the significance of the increased CUL3-KEAP1 interaction is currently not clear.

### Investigating novel NRF2 inducing compounds in endophytes

In our described work above, we characterized the NRF2 inducer andrographolide, which is isolated from the medicinal plant *Andrographis paniculata*. NRF2 is also induced by various other plant derived compounds. Here, we sought to investigate plant endophytes for bioactive metabolites with NRF2 inducing properties. We focused specifically on non-flowering plants that are local to Singapore. Among the plants chosen were six species of ferns and four species of mosses (see Materials and Methods).

### Investigation of the effects of organic extracts isolated from the endophytes on NRF2 transcriptional activity

A total of 49 species of bacterial and fungal endophytes were isolated (ORX 1 to ORX 49; data not shown). To test if the secondary metabolites produced by the endophytes had NRF2-inducing properties, the secondary metabolites were extracted using dichloromethane as described under Materials and Methods. The resultant organic extracts contain bioactive metabolites that have diffused from the bacterial and fungal endophytes into the dichloromethane solvent. The organic extracts were dissolved in methanol and used for further testing.

**Fig 6:**
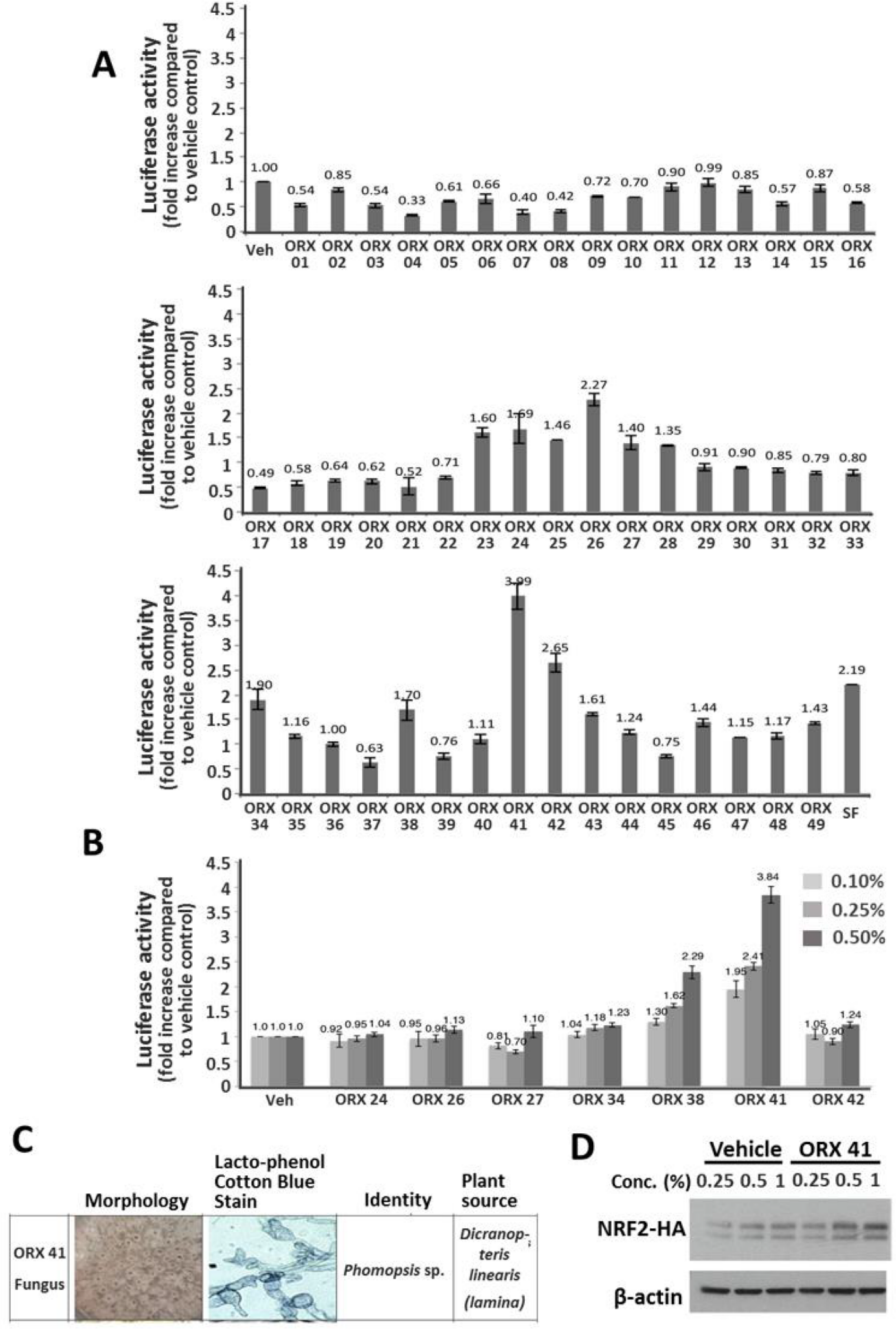
Effects of organic extracts isolated from the endophytes on NRF2. (A) To screen the 49 organic extracts isolated from the endophytes for NRF2 transcriptional activation, the ARE reporter assay was performed as described under Materials and Methods. HEK293T cells were transfected with ARE-pGL2 plasmid for 24 hours before treatment with the organic extracts (0.5%) or sulforaphane (SF; 10 μM) for six hours before analysis. (B) Dose response of the effect of organic extracts on NRF2 transcriptional activation. The assay was carried out as in (A). (C) Morphology, Lacto-phenol Cotton Blue staining and identity (based on ≥99% sequence similarity of BLAST results) of the fungal endophyte from which ORX 41 was derived. (D) Western blot analysis of the effect of organic extract ORX 41 on the NRF2 protein concentration. HEK293T cells were transfected with HA-NRF2 for 2 days before treatment with vehicle (methanol treatment) or ORX 41 for 6 hours at the indicated concentrations, followed by Western blotting with HA and β-actin antibody.

We screened the 49 organic extracts for NRF2-inducing properties using the antioxidant response elements (ARE) dependent luciferase reporter assay. As shown in Fig 6A, 18 out of the 49 organic extracts showed an increase in NRF2 transcription activity (fold increase >1 compared to vehicle control). Notably, the extract ORX 41 showed the highest increase in NRF2 transcriptional activity compared to the vehicle control (3.99 fold) (Fig 6A).

We next performed a dose response ARE gene reporter assay with seven of the most promising organic extracts. As shown in Fig 6B, treatment with ORX 41 caused the greatest increase in NRF2 transcriptional activity and this effect was dose dependent. ORX 41 was derived from a fungus species of the genus *Phomopsis*, isolated from the lamina of the plant *Dicranopteris linearis* (Fig 6C).

Since NRF2 is regulated primarily via its protein stability, we sought to investigate the effect of the organic extract ORX 41 on NRF2 protein levels. As shown in Fig 5D, cells treated with ORX 41 showed an increase in the NRF2 protein concentration compared to control cells. The effect of ORX 41 was dose dependent. Hence, we conclude that organic extract ORX 41 has NRF2 inducing activity.

## Discussion

In this study, we have confirmed that andrographolide is an NRF2 inducer and provided evidence that at low concentrations, the drug acts by inhibiting the function of the CUL3-RBX1-KEAP1 E3 ubiquitin ligase. We also showed that the effect of low andrographolide concentrations is dependent on Cys151 in KEAP1. Similar to other compounds that act via Cys151, andrographolide may function by disrupting the interaction between KEAP1 and CUL3. We have also shown that at higher concentrations, andrographolide acts via a mechanism independent of Cys151 in KEAP1. The effect of high andrographolide concentrations may be due to the interaction of the drug with other cysteine residues in KEAP1. Alternatively, andrographolide may potentially induce NRF2 protein expression in a KEAP1 independent manner.

In previous studies, Yen et al., 2016 and Wong et al., 2016 found that induction of the NRF2 protein and the NRF2 transcriptional target HO-1 by andrographolide is partially prevented by treatment with the p38 MAPK inhibitor SB203580. A similar, though less pronounced inhibitory effect NRF2 protein expression and HO-1 mRNA expression was observed with the ERK inhibitor PD98059 [6]. In these two studies, the authors used andrographolide concentrations of 10 μM and 30 μM, respectively [6, 7]. These concentrations are similar to the low concentration of 7.5 μM used in our study. The reasons for the discrepant results are currently unclear. However, it is important to note that the previous studies observed that the p38 and ERK inhibitors blocked the andrographolide dependent increase in NRF2 protein expression only partially. In contrast, we have shown that mutating Cys151 in Keap1 completely prevents the effect of 7.5 μM andrographolide. One caveat is that in our studies we relied on overexpressed proteins, as it was necessary to exogenously introduce the C151S KEAP1 mutant into cells. Nonetheless, in support of a Cys151 dependent mechanism of action of andrographolide, Yuan et al., 2012 have reported that andrographolide can indeed form an adduct with Cys151 of recombinant KEAP1 *in vitro*.

What is the mechanism through which compounds that interact with KEAP1 lead to inhibition of NRF2 ubiquitination? Various NRF2 stabilizing compounds form adducts with Cys151 in KEAP1. Cys151 localises to the KEAP1 BTB domain, which mediates KEAP1 homodimerization as well as binding to the E3 ubiquitin ligase scaffold protein CUL3. It has been suggested that drugs that bind to Cys151 lead to a dissociation of CUL3 from the KEAP1 substrate receptor [10–14]. On the other hand, it has also been shown that NRF2 stabilization by Cys151 dependent NRF2 inducers can occur in the absence if CUL3-KEAP1 dissociation and is accompanied by a conformational change in the KEAP1 protein [23]. Of note, our results indicate that mediating binding to CUL3 is unlikely to be the only function of the KEAP1 BTB domain. Thus, when we substituted the Keap1 BTB domain with that of a different CUL3 substrate receptor protein (KLHL12), BTB homodimerization and binding to CUL3 were preserved, but NRF2 degradation was prevented. This confirms that the BTB domain also exerts conformational control over the E3 ubiquitin ligase complex, which may be disrupted by Cys151 interacting drugs [24].

In addition to Cys151, Yuan et al., 2012 have also shown that andrographolide reacts with Cys77, Cys273 and Cys368 residues in KEAP1 *in vitro*. This suggests that andrographolide can potentially inhibit KEAP1 function through the interaction with other cysteine residues. We indeed observed that at a high concentration of 100 μM, andrographolide induces NRF2 protein expression in a Cys151 independent manner. At this concentration, andrographolide increased the binding of both wild type and C151S mutant KEAP1 to CUL3, suggesting that the effect of andrographolide at this concentration is mediated through Keap1 as well. Of note, other NRF2 inducers that stabilize the transcription factor in a Cys151 independent manner, e.g. arsenite, monomethylarsonous, PMX290 and nitro-fatty acids, have also been reported to cause an increase in the interaction between KEAP1 and CUL3 [19, 21, 25]. It is likely that these NRF2 inducers cause a conformational change in KEAP1 that is accompanied by an increased interaction between KEAP1 and CUL3. However, whether the increased KEAP1-CUL3 binding is functionally significant is currently not clear. Further elucidation of the underlying mechanism will improve our understanding of Keap1 function and may aid future drug design for chemopreventive and therapeutic purposes.

To identify novel NRF2 activating compounds, we also isolated endophytes from non-flowering plants local to Singapore and tested whether the endophytes produce bioactive metabolites with NRF2 inducing properties. We focussed on tropical ferns and mosses, some of which have been traditionally used in many countries in Asia as traditional medicine remedies to treat various conditions. Examples include the use of *Phlegmariurus phlegmaria (*also known as ‘coarse tassel fern’) applied to wounds in the form of a paste [26] or the use of *Taenitis blechnoides* as medicine for post-natal women in native tribes in Malaysia and in India [27, 28]. Apart from these non-flowering plants, *Psilotum nudum* [29, 30] and *Dicranopteris linearis* [31] have also been suggested to have antioxidant properties. Thus, given that non-flowering plants have been implicated to have anti-inflammatory and antioxidant properties, we hypothesized that the fungal and bacterial endophytes living within these plants may also produce secondary bioactive metabolites with NRF2 inducing properties.

Our results show that among the organic extracts isolated from the bacterial and fungal endophytes, ORX 41 is the most promising extract as it showed significant NRF2-inducing properties. ORX 41 is the dichloromethane extract of a *Phomopsis* sp., a fungal endophyte isolated from the lamina of *Dicranopteris linearis*. Interestingly, a recent study reported that the acetonitrile extract of the fungal endophyte *Phomopsis* sp.Cs-c2 from *Senna spectabilis* (DC.) contains a number of compounds, cytochalasin H, cytochalasin J and alternariol, with anti-inflammatory properties [32]. The compounds were shown to inhibit the production of reactive oxygen species by stimulated neutrophils. It would be interesting to test whether these compounds are present in our dichloromethane extract and whether the compounds have NRF2 inducing properties.

Hence, we conclude that ORX 41 has the greatest potential in inducing NRF2. Further studies are needed to identify the exact component within this crude organic extract that accounts for its NRF2 inducing properties. The identification and characterization of the NRF2 inducing compound within the organic extract ORX 41 could lead to the discovery of a novel NRF2 inducer.

## Supporting information

S1 Fig Western blot analysis of NRF2 protein concentrations in HEK293T cells after treatment with andrographolide and arsenite. (A, B) HEK293T cells were transfected with HA-NRF2 and either wild type FLAG-KEAP1 or C151S mutant FLAG-KEAP1 for 2 days before treatment with andrographolide (7.5 μM) and arsenite (20 μM) for 6 hours. The cell lysate samples were subjected to SDS-PAGE and analyzed using Western Blotting with HA and FLAG antibodies.
(TIF)

S2 Fig Binding of KEAP1 mutants, deletions and chimeric proteins to CUL3, KEAP1 (via homodimerization) and NRF2. (A, B) HEK293T cells were transfected with the indicated expression plasmids for 2 days. The cells were then lysed and subjected to immunoprecipitation with FLAG M2 agarose. Cell lysates and immunoprecipitates were analyzed by Western blotting with HA and FLAG antibodies. Details of the used expression plasmids are included in Materials and Methods.
(TIF)

S3 Fig Mutation of Cys273 and Cys288 does not affect KEAP1 subcellular localization. HEK293T cells were transfected with the indicated expression plasmids for 2 days, followed by immunofluorescence staining using FLAG M2 antibody. For immunofluorescence staining, the cells were fixed using 4% paraformaldehyde, permeabilized with triton 0.1% triton X-100 and, after blocking with 5% fetal bovine serum, incubated with FLAG primary antibody and secondary FITC-conjugated anti-mouse IgG.
(TIF)

S4 Fig Effect of andrographolide on the interaction between KEAP1 and CUL3. The cells were transfected with the indicated expression plasmids for two days, followed by drug treatment with 7.5 μM andrographolide for 4 hours.
(TIF)

## References

[1] WHO Monographs on Selected Medicinal Plants 2002, World Health Organization: Geneva, Switzerland 12–24.

[2] Lim JCW, Chan TK, Ng DS, Sagineedu SR, Stanslas J, and Wong W, Andrographolide and its analogues: versatile bioactive molecules for combating inflammation and cancer. Clin. Exp. Pharmacol. Physiol., 2012 39(3): p. 300–310. 10.1111/j.1440-1681.2011.05633.x 22017767

[3] Yang C-H, Yen T-L, Hsu C-Y, Thomas P-A, Sheu J-R, and Jayakumar T, Multi-targeting andrographolide, a novel NF-κB inhibitor, as a potential therapeutic agent for stroke. Int. J. Mol. Sci., 2017 18(8): p. 1638. 10.3390/ijms18081638 PMC5578028 28749412

[4] Xia Y-F, Ye B-Q, Li Y-D, Wang J-G, He X-J, Lin X, et al, Andrographolide attenuates inflammation by inhibition of NF-κB activation through covalent modification of reduced cysteine 62 of p50. Journal Immunol, 2004 173(6): p. 4207–4217. 15356172 10.4049/jimmunol.173.6.4207

[5] Tan WSD, Liao W, Zhou S, and Wong WF, Is there a future for andrographolide to be an anti-inflammatory drug? Deciphering its major mechanisms of action. Biochem. Pharmacol., 2017 139: p. 71–81. 10.1016/j.bcp.2017.03.024 28377280

[6] Wong SY, Tan MG, Wong PT, Herr DR, and Lai MK, Andrographolide induces Nrf2 and heme oxygenase 1 in astrocytes by activating p38 MAPK and ERK. J. Neuroinflammation, 2016 13(1): p. 251 10.1186/s12974-016-0723-3 27663973 PMC5034653

[7] Yen T-L, Chen R-J, Jayakumar T, Lu W-J, Hsieh C-Y, Hsu M-J, et al, Andrographolide stimulates p38 mitogen-activated protein kinase–nuclear factor erythroid-2-related factor 2–heme oxygenase 1 signaling in primary cerebral endothelial cells for definite protection against ischemic stroke in rats. Transl. Res., 2016 170: p. 57–72. 10.1016/j.trsl.2015.12.002 26746802

[8] Yuan Y, Ji L, Luo L, Lu J, Ma X, Ma Z, et al, Quinone reductase (QR) inducers from Andrographis paniculata and identification of molecular target of andrographolide. Fitoterapia, 2012 83(8): p. 1506–1513. 10.1016/j.fitote.2012.08.018 22960348

[9] Taguchi K, Motohashi H, and Yamamoto M, Molecular mechanisms of the Keap1–Nrf2 pathway in stress response and cancer evolution. Genes Cells, 2011 16(2): p. 123–140. 10.1111/j.1365-2443.2010.01473.x 21251164

[10] Zhang DD, Lo S-C, Cross JV, Templeton DJ, and Hannink M, Keap1 is a redox-regulated substrate adaptor protein for a Cul3-dependent ubiquitin ligase complex. Mol. Cell. Biol., 2004 24(24): p. 10941–10953. 10.1128/MCB.24.24.10941-10953.2004 15572695 PMC533977

[11] Rachakonda G, Xiong Y, Sekhar KR, Stamer SL, Liebler DC, and Freeman ML, Covalent modification at Cys151 dissociates the electrophile sensor Keap1 from the ubiquitin ligase CUL3. Chem. Res. Toxicol., 2008 21(3): p. 705–710. 10.1021/tx700302s 18251510

[12] Eggler AL, Small E, Hannink M, and Mesecar AD, Cul3-mediated Nrf2 ubiquitination and antioxidant response element (ARE) activation are dependent on the partial molar volume at position 151 of Keap1. Biochem. J., 2009 422(1): p. 171–180. 10.1042/BJ20090471 19489739 PMC3865926

[13] Ichikawa T, Li J, Meyer CJ, Janicki JS, Hannink M, and Cui T, Dihydro-CDDO-trifluoroethyl amide (dh404), a novel Nrf2 activator, suppresses oxidative stress in cardiomyocytes. PLoS One, 2009 4(12): p. e8391 10.1371/journal.pone.0008391 20027226 PMC2791441

[14] Cleasby A, Yon J, Day PJ, Richardson C, Tickle IJ, Williams PA, et al, Structure of the BTB domain of Keap1 and its interaction with the triterpenoid antagonist CDDO. PLoS One, 2014 9(6): p. e98896 10.1371/journal.pone.0098896 24896564 PMC4045772

[15] Stefanson AL and Bakovic M, Dietary regulation of Keap1/Nrf2/ARE pathway: focus on plant-derived compounds and trace minerals. Nutrients, 2014 6(9): p. 3777–3801. 10.3390/nu6093777 25244368 PMC4179188

[16] Zhao J, Shan T, Mou Y, and Zhou L, Plant-derived bioactive compounds produced by endophytic fungi. Mini Rev. Med. Chem., 2011 11(2): p. 159–168. 21222580 10.2174/138955711794519492

[17] Bascom-Slack CA, Arnold AE, and Strobel SA, Student-directed discovery of the plant microbiome and its products. Science, 2012 338(6106): p. 485–486. 10.1126/science.1215227 23112324

[18] Chew E-H and Hagen T, Substrate-mediated regulation of cullin neddylation. J. Biol. Chem., 2007 282(23): p. 17032–17040. 10.1074/jbc.M701153200 17439941

[19] Wong DPW, Wells G, and Hagen T, Heteroaromatic 4-arylquinols are novel inducers of nuclear factor-erythroid 2-related factor 2 (Nrf2). Eur. J. Pharmacol., 2010 643(2–3): p. 188–194. 10.1016/j.ejphar.2010.06.040 20599909

[20] Dhakshinamoorthy S and Porter AG, Nitric oxide-induced transcriptional up-regulation of protective genes by Nrf2 via the antioxidant response element counteracts apoptosis of neuroblastoma cells. J. Biol. Chem., 2004 279(19): p. 20096–20107. 10.1074/jbc.M312492200 14985350

[21] Wang X-J, Sun Z, Chen W, Li Y, Villeneuve NF, and Zhang DD, Activation of Nrf2 by arsenite and monomethylarsonous acid is independent of Keap1-C151: enhanced Keap1–Cul3 interaction. Toxicol. Appl. Pharmacol., 2008 230(3): p. 383–389. 10.1016/j.taap.2008.03.003 18417180 PMC2610481

[22] Zhang DD and Hannink M, Distinct cysteine residues in Keap1 are required for Keap1-dependent ubiquitination of Nrf2 and for stabilization of Nrf2 by chemopreventive agents and oxidative stress. Mol. Cell. Biol., 2003 23(22): p. 8137–8151. 10.1128/MCB.23.22.8137-8151.2003 14585973 PMC262403

[23] Baird L and Dinkova-Kostova AT, Diffusion dynamics of the Keap1–Cullin3 interaction in single live cells. Biochem. Biophys. Res. Commun., 2013 433(1): p. 58–65. 10.1016/j.bbrc.2013.02.065 23454126

[24] Baird L, Llères D, Swift S, and Dinkova-Kostova AT, Regulatory flexibility in the Nrf2-mediated stress response is conferred by conformational cycling of the Keap1-Nrf2 protein complex. Proc Natl Acad Sci USA, 2013 110(38): p. 15259–15264. 10.1073/pnas.1305687110 23986495 PMC3780858

[25] Kansanen E, Bonacci G, Schopfer FJ, Kuosmanen SM, Tong KI, Leinonen H, et al, Electrophilic nitro-fatty acids activate NRF2 by a KEAP1 cysteine 151-independent mechanism. J. Biol. Chem., 2011 286(16): p. 14019–14027. 10.1074/jbc.M110.190710 21357422 PMC3077602

[26] Revathi RM R, Binu Thomas K. Ethno medicinal fern and fernallies used by tribe Malayalis of Kolli Hills, Eastern Ghats. Pteridol Res, 2013 2(1): p. 1–10.

[27] Trivedi PC, Medicinal plants: utilisation and conservation. 2009: Aavishkar Publishers, Distributors.

[28] Eswani N, Kudus KA, Nazre M, Noor AA, and Ali M, Medicinal plant diversity and vegetation analysis of logged over hill forest of Tekai Tembeling Forest Reserve, Jerantut, Pahang. J Agric Sci, 2010 2(3): p. 189.

[29] Cooper-Driver G, Chemical evidence for separating the Psilotaceae from the Filicales. Science, 1977 198(4323): p. 1260–1262. 10.1126/science.198.4323.1260 17741707

[30] Li X, Wang L, Han W, Mai W, Han L, and Chen D, Amentoflavone protects against hydroxyl radical-induced DNA damage via antioxidant mechanism. Turk J Biochem, 2014 39(1): p. 30–36.

[31] Zakaria Z, Mohamed A, Jamil NM, Rofiee M, Somchit M, Zuraini A, et al, In vitro cytotoxic and antioxidant properties of the aqueous, chloroform and methanol extracts of *Dicranopteris linearis* leaves. Afr J Biotechnol, 2011 10(2): p. 273–282.

[32] Chapla VM, Zeraik ML, Ximenes VF, Zanardi LM, Lopes MN, Cavalheiro AJ, et al, Bioactive secondary metabolites from *Phomopsis* sp., an endophytic fungus from *Senna spectabilis*. Molecules, 2014 19(5): p. 6597–6608. 10.3390/molecules19056597 24858094 PMC6271730

