# Supplementary figures and images for: Regulation of the NRF2 transcription factor by andrographolide and organic extracts from plant endophytes
Source: PLoS One. 2018 Oct 1;13(10):e0204853. doi: 10.1371/journal.pone.0204853 (PMC6166955; doi:10.1371/journal.pone.0204853)

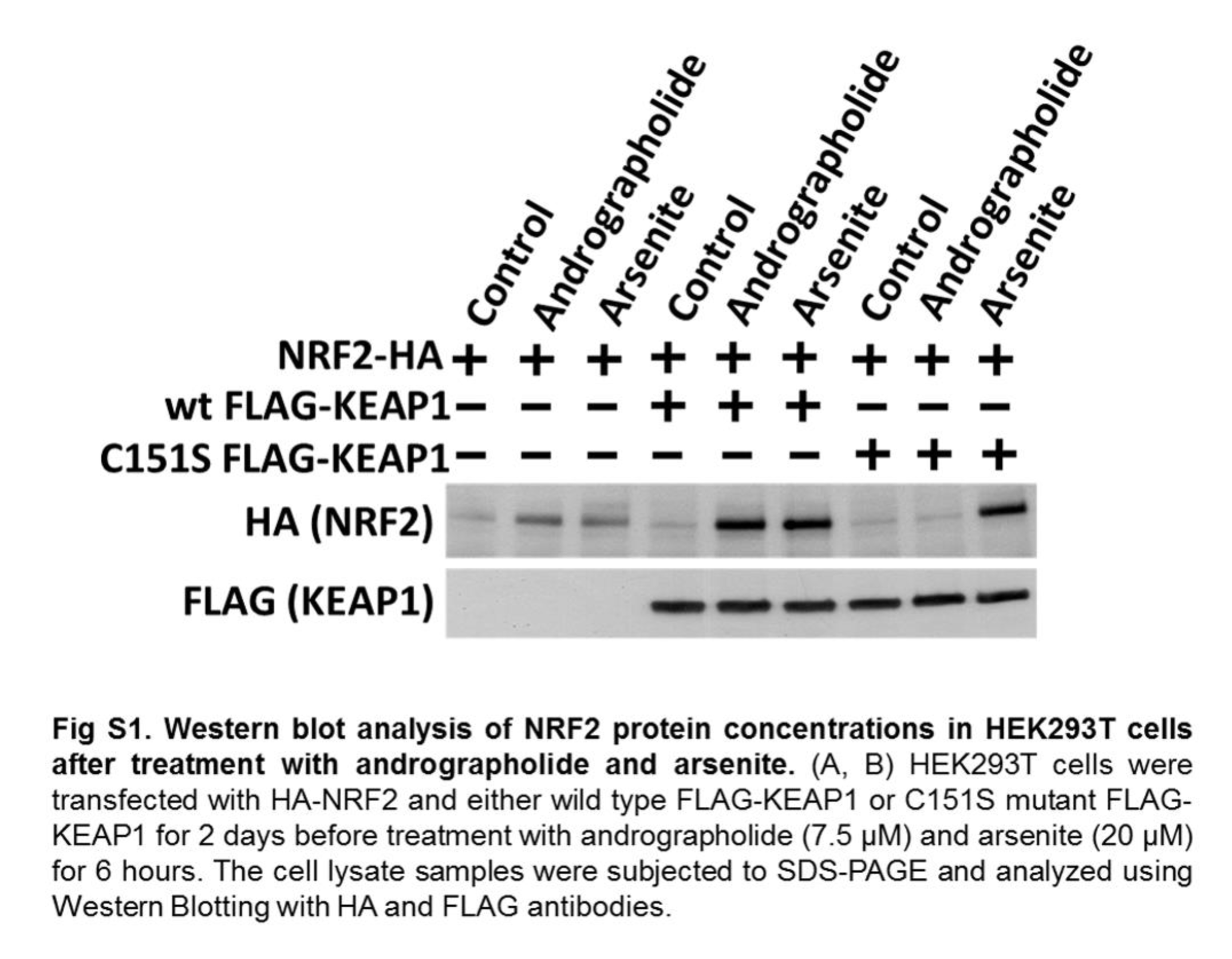

Supplement: S1 Fig — (A, B) HEK293T cells were transfected with HA-NRF2 and either wild type FLAG-KEAP1 or C151S mutant FLAG-KEAP1 for 2 days before treatment with andrographolide (7.5 μM) and arsenite (20 μM) for 6 hours. The cell lysate samples were subjected to SDS-PAGE and analyzed using Western Blotting with HA and FLAG antibodies. (TIF) [file pone.0204853.s001.tif]

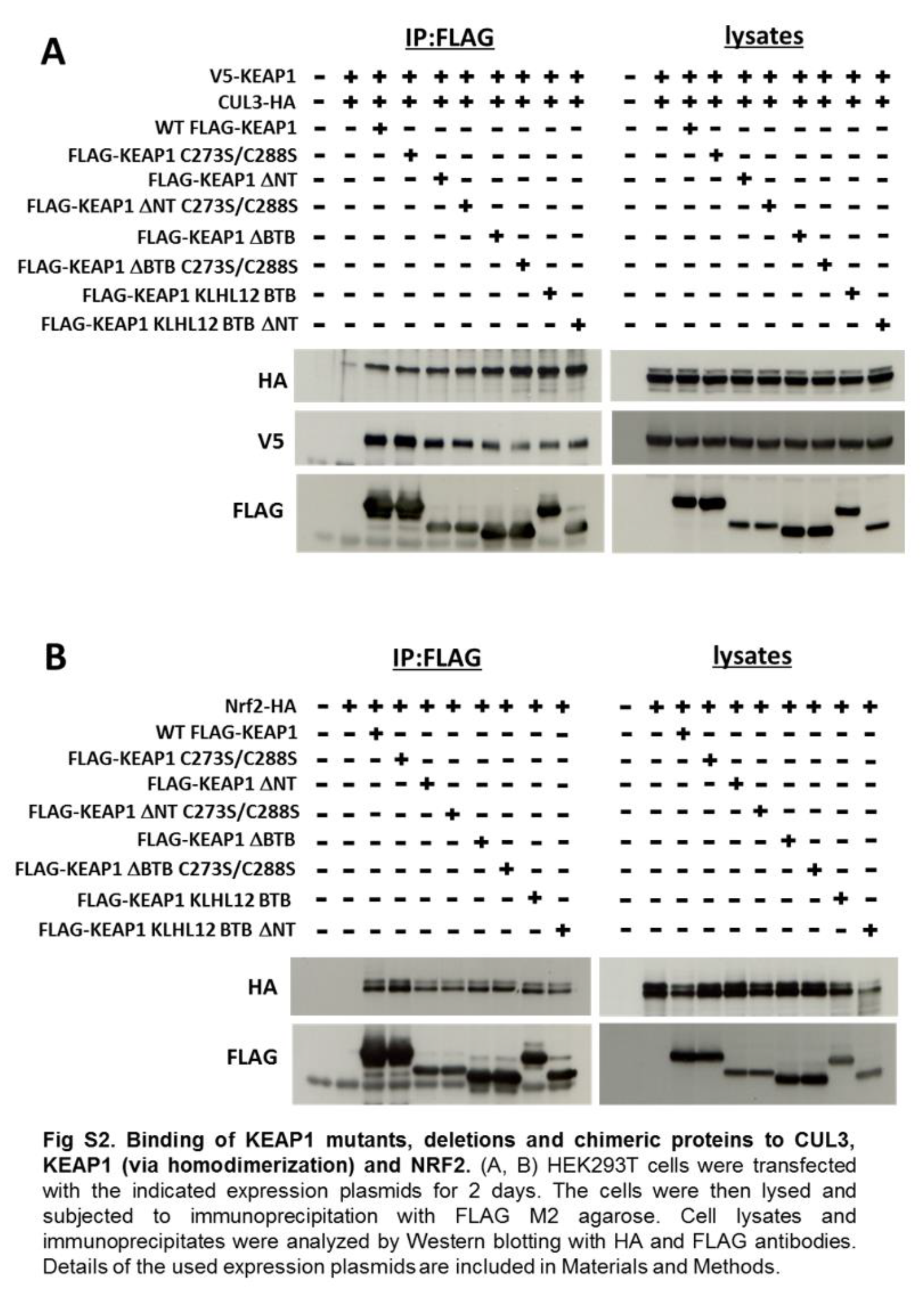

Supplement: S2 Fig — (A, B) HEK293T cells were transfected with the indicated expression plasmids for 2 days. The cells were then lysed and subjected to immunoprecipitation with FLAG M2 agarose. Cell lysates and immunoprecipitates were analyzed by Western blotting with HA and FLAG antibodies. Details of the used expression plasmids are included in Materials and Methods. (TIF) [file pone.0204853.s002.tif]

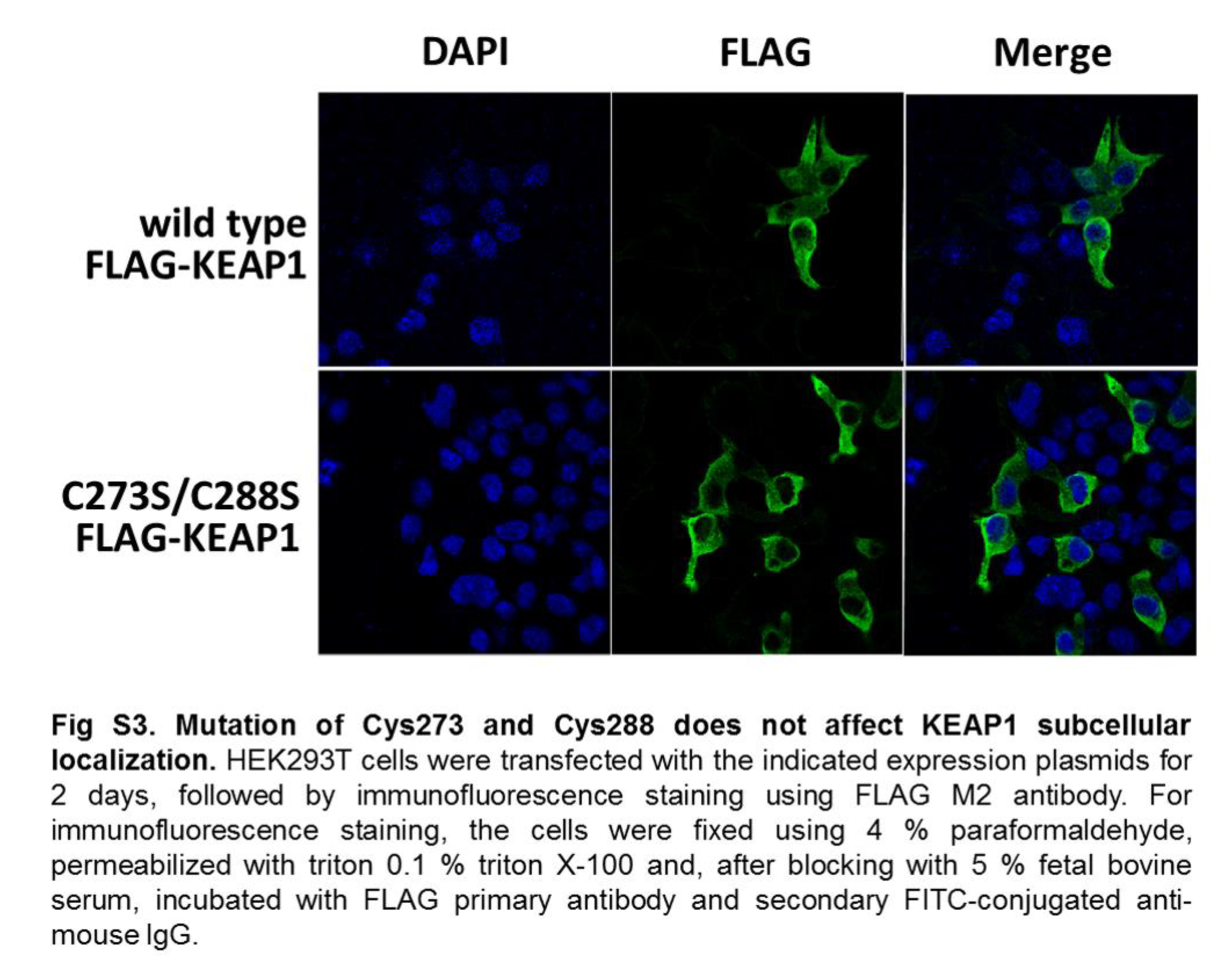

Supplement: S3 Fig — HEK293T cells were transfected with the indicated expression plasmids for 2 days, followed by immunofluorescence staining using FLAG M2 antibody. For immunofluorescence staining, the cells were fixed using 4% paraformaldehyde, permeabilized with triton 0.1% triton X-100 and, after blocking with 5% fetal bovine serum, incubated with FLAG primary antibody and secondary FITC-conjugated anti-mouse IgG. (TIF) [file pone.0204853.s003.tif]

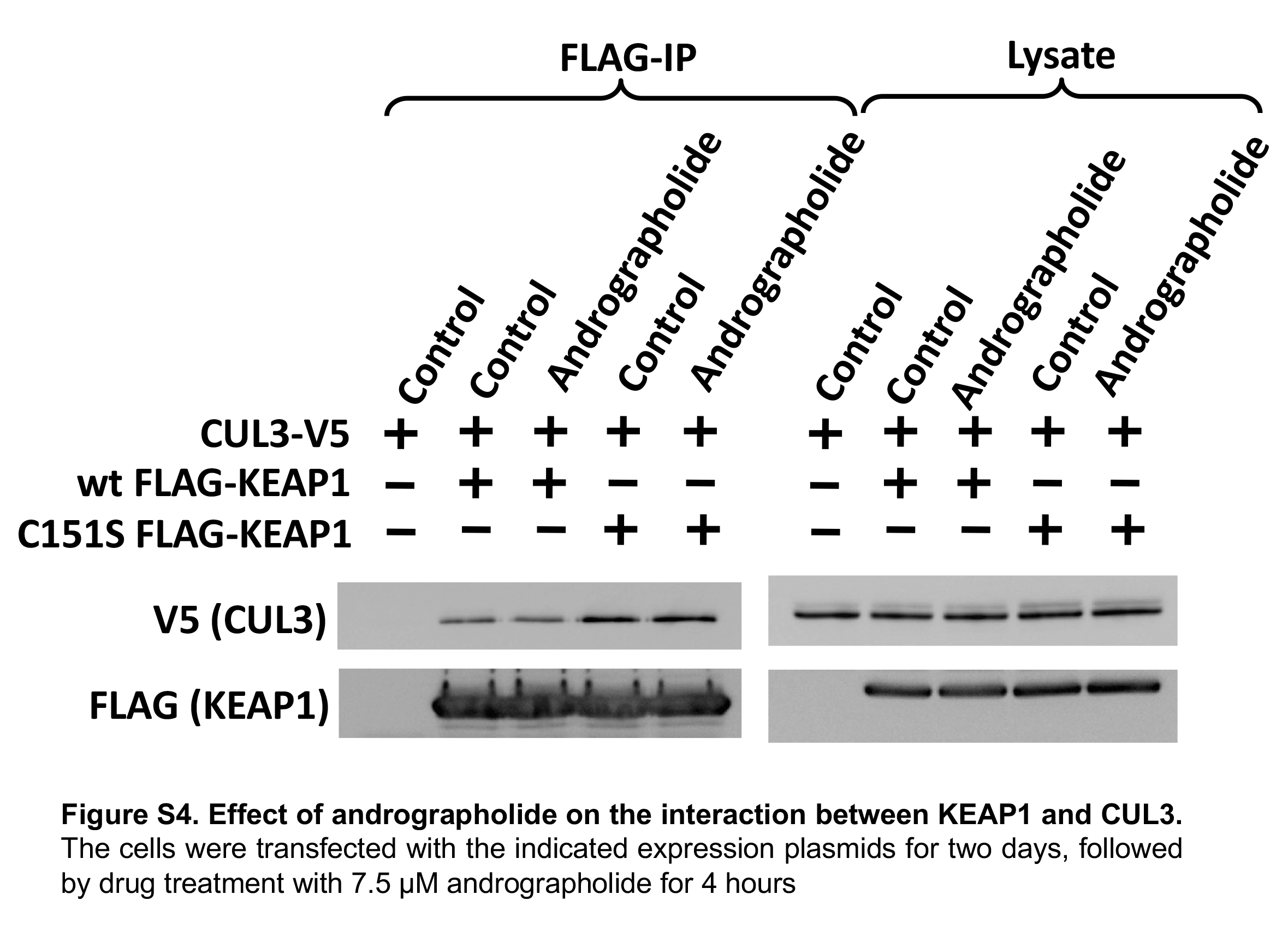

Supplement: S4 Fig — The cells were transfected with the indicated expression plasmids for two days, followed by drug treatment with 7.5 μM andrographolide for 4 hours. (TIF) [file pone.0204853.s004.tif]
